# Improving quality of life in patients with rare autoimmune liver diseases by structured peer-delivered support (Q.RARE.LI): study protocol for a transnational effectiveness-implementation hybrid trial

**DOI:** 10.1186/s12888-023-04669-0

**Published:** 2023-03-24

**Authors:** Natalie Uhlenbusch, Arpinder Bal, Boglárka Balogh, Annika Braun, Anja Geerts, Gideon Hirschfield, Maciej K. Janik, Ansgar W. Lohse, Piotr Milkiewicz, Mária Papp, Carine Poppe, Christoph Schramm, Bernd Löwe

**Affiliations:** 1 Department of Psychosomatic Medicine and Psychotherapy, University Medical Centre Hamburg-Eppendorf, Hamburg, Germany; 2 Toronto Centre for Liver Disease, University Health Network, Toronto, Canada; 3 Department of Internal Medicine, Division of Gastroenterology, Faculty of Medicine, University of Debrecen, Debrecen, Hungary; 4 Department of Internal Medicine and Pediatrics, Ghent University Hospital, Ghent, Belgium; 5 Department of Hepatology, Transplantology and Internal Medicine, Medical University of Warsaw, Warsaw, Poland; 6 Department of Medicine, University Medical Centre Hamburg-Eppendorf, Hamburg, Germany; 7 Translational Medicine Group, Pomeranian Medical University, Pomeranian, Poland; 8 Martin Zeitz Center for Rare Diseases, University Medical Centre Hamburg-Eppendorf, Hamburg, Germany

**Keywords:** Quality of life, Rare diseases, Liver diseases, Effectiveness-implementation hybrid trial, Psychosocial support, Peer-support, Psychosomatic medicine

## Abstract

**Background:**

Psychosocial support is a crucial component of adequate rare disease care, but to date psychosocial support needs of this patient population are insufficiently met. Within Q.RARE.LI, we strive to evaluate the effectiveness of a structured, transdiagnostic, and location-independent psychosocial support intervention in routine care of patients with rare autoimmune liver diseases in five countries and prepare its implementation.

**Methods:**

Within an effectiveness-implementation hybrid trial, we aim to a) investigate the effectiveness of the intervention in routine care in five diverse healthcare systems and b) assess implementation outcomes, examine and prepare the implementation context, and develop country-specific implementation strategies. To assess effectiveness, we will include *N* = 240 patients with rare autoimmune liver diseases. Within a two-armed randomized controlled trial (allocation ratio 1:1), we will compare structured and peer-delivered psychosocial support in addition to care-as-usual (CAU) with CAU alone. Outcomes will be assessed via electronic database entry prior to intervention, directly after, and at a three-month follow-up. Our primary effectiveness outcome will be mental health-related quality of life at post-assessment. Secondary outcomes include depression and anxiety severity, perceived social support, helplessness, and disease acceptance. Implementation outcomes will be assessed within a mixed-methods process evaluation. In a quantitative cross-sectional survey, we will examine perceived acceptability and feasibility in patients, peer-counselors, and healthcare providers involved in delivery of the intervention. In qualitative focus groups, we will analyze the implementation context and determine barriers and facilitators for implementation with different stakeholders (patients and/or representatives, peer-counselors, healthcare providers, health insurers). Based on these results, we will derive country-specific implementation strategies and develop a concrete implementation plan for each country.

**Discussion:**

The intervention is expected to help patients adjust to their disease and improve their mental quality of life. The transdiagnostic and location-independent program has the potential to reach patients for psychosocial support who are usually hard to reach. By preparing the implementation in five countries, the project can help to make low-threshold psychosocial support available to many patients with rare diseases and improve comprehensive healthcare for an often neglected group.

**Trial registration:**

ISRCTN15030282

## Background

Rare diseases per definition appear in less than one in 2000 individuals. With each rare disease having a low prevalence and a worldwide very high number of different rare diseases (~ 6000), research, healthcare, and affected individuals are confronted with a variety of challenges. In many cases, knowledge about the diseases is scarce, diagnostic processes are long and difficult, and adequate treatment is limited. Patients often are geographically dispersed, lack contact to peers, and have to travel long distances to reach specialized care [1–3]. These rarity-specific aspects add to the burden that most patients with chronic conditions face, such as persistent somatic suffering or constraints in everyday life. The majority of rare diseases are genetically caused, progressive, and mostly there is no cure available, confronting individuals with the lifelong task of adjusting to their condition. How challenging this can be, is indicated by increased rates of depressive and anxiety symptoms [4] and disorders [5] across different rare conditions. Psychological health is a key determinant for quality of life. In absence of a cure for most rare diseases, improving quality of life is a major healthcare aim. Therefore, supporting patients in adjusting to their diseases and staying mentally healthy is crucial. In a systematic review on quality of life in rare diseases, Cohen and Biesecker (2010) found that quality of life is often reduced in this patient group [6]. However, this is not necessarily the case, depending on whether disease adjustment succeeds. The authors conclude that psychological support can be essential for patients’ overall wellbeing.

To date, psychosocial support needs of patients with rare diseases are not sufficiently met [7, 8]. Patients wish for more support in different areas of their life, such as physical support and daily living, support concerning the health system and information, or psychological support [7]. Moreover, many patients do not feel sufficiently socially supported and lack contact to peers with the same condition [1, 9]. Support programs that are available for patients with common chronic conditions, such as diabetes [10] or COPD [11], may not adequately address the unique challenges patients with rare diseases face [12]. In addition, disease-specific approaches are not feasible due to the high number of different rare diseases [13]. Geographical dispersion of patients further complicates reaching individuals with rare diseases for healthcare services.

To tackle these challenges and address the unmet psychosocial support needs of the rare disease community, we developed a support program at the University Medical Center Hamburg-Eppendorf in Germany. The program was developed based on pre-assessed needs of patients with rare diseases [14]. Patients receive a structured self-help manual, which they complete from home over the course of six weeks. Once a week, they receive telephone-based support by a trained and supervised peer-counselor to reflect on the content of the self-management book. The program was evaluated within a monocentric two-armed randomized efficacy trial including *N* = 89 patients with four heterogeneous rare diseases [15]. The program was highly accepted by patients and demonstrated efficacy regarding mental health-related quality of life, disease acceptance, coping abilities, perceived social support, and helplessness. The program has the potential to address the unmet support needs of patients with rare diseases. It has been conceptualized transdiagnostically, focusing on the common experiences of patients with different conditions [9]. It is location-independent and therefore tackles the challenge that patients are often geographically dispersed. It is peer-delivered in order to address the need of patients to connect with each other. However, it is not yet available to patients, which is the starting point for Q.RARE.LI. As it is not feasible to provide specific psychosocial interventions for every single rare disease, we pursue a transdiagnostic approach focusing on the common needs of patients with rare diseases. One way to do so is by building subgroups, for instance based on the affected organ system [12, 16]. We aim to make the support program available to the rare disease community, starting with rare autoimmune liver diseases.

Rare autoimmune liver diseases include autoimmune hepatitis (AIH, prevalence 0.5–1/100,000), primary sclerosing cholangitis (PSC, prevalence 1–9/100,000) and primary biliary cholangitis (PBC, prevalence 1–5/10,000). All three diseases have an autoimmune and cholestatic etiology, are incurable and progressive, and go along with clinical symptom burden such as damage to the liver or chronic fatigue. The challenges patients with rare liver diseases face reflect those of the wider rare disease community (difficulties in diagnosis, lack of information, isolation, uncertainty, stigmatization [17]) and the need for psychosocial support is particularly high, as indicated by reduced quality of life [18–23] and high psychopathological burden [24, 25].

### Objectives and hypotheses

The overall aim of Q.RARE.LI is to make a structured, peer-delivered psychosocial support program available to patients with rare autoimmune liver diseases. More specifically, we aim to evaluate the effectiveness of the program in routine care of five different healthcare settings. In addition, we aim to prepare the implementation into routine care by assessing implementation outcomes and developing implementation strategies in each of the five participating countries. Regarding the effectiveness, we hypothesize that structured, peer-delivered support in addition to care-as-usual (CAU) leads to 1) an improvement in mental health-related quality of life and 2) improved outcomes regarding a) physical health-related quality of life, b) depression severity, c) anxiety severity, d) illness acceptance, e) perceived helplessness, f) social support, and g) self-management abilities in patients with rare liver diseases compared to CAU alone. Concerning implementation outcomes, we hypothesize that the program shows high feasibility and acceptability as indicated by 1) > 75% of the patients completing the intervention, 2) > 75% of these rating the intervention as a) helpful and b) appropriate, 3) 75% of the stakeholders delivering the intervention rating it as a) feasible, b) appropriate, and c) helpful for patients.

## Methods

This study protocol follows the *Standard Protocol Item: Recommendations for Interventional Trial* (SPIRIT) guidelines (spirit-statement.org). The trial has been registered at https://doi.org/10.1186/ISRCTN15030282. If any important modifications from the protocol are made, the protocol will be updated there. Q.RARE.LI is funded by the *Joint Transnational Call 2021* within the *European Joint Programme on Rare Diseases*.

### Trial design

To ensure the dual focus of investigating both effectiveness and implementability, we chose an effectiveness-implementation hybrid trial design [26]. These are study designs blending the processes of effectiveness and implementation research. This can accelerate the translation of research into clinical practice [26], and helps to better understand the contextual factors related to the success of interventions [27]. We will conduct an effectiveness-implementation hybrid trial type 1 [26] with the primary aim of assessing the effectiveness of the intervention while also analyzing its implementability.

**Fig. 1:**
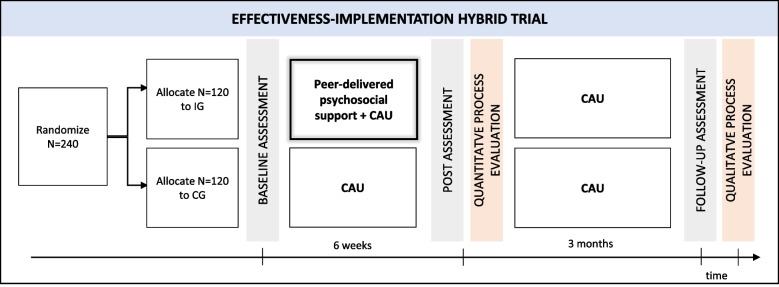
Study design. *Notes*. IG = intervention group, CG = control group, CAU = care-as-usual

To investigate effectiveness, we will conduct a two-armed randomized superiority trial. Within a parallel group design, patients with rare autoimmune liver diseases will be randomly assigned to either the intervention or a control group with a 1:1 allocation ratio. To evaluate implementability, we will conduct a mixed-method process evaluation assessing implementation outcomes both quantitatively (cross-sectional survey) and qualitatively (focus groups). The quantitative data enables us to measure how well the intervention was accepted and how feasible its implementation is perceived, and ensures a clear conclusion to our formulated hypothesis. The qualitative results will complement these data by helping to gain a deeper understanding of how well applying the intervention worked in routine care, and what facilitates and hinders a successful implementation from the perspective of different stakeholders (e.g. patient representatives, healthcare providers). The qualitative data further will help to develop concrete implementation strategies. Figure**1** shows an overview of the study design.

### Study setting

The study will be conducted in routine care of patients with rare autoimmune liver diseases at five different centers: The University Medical Center Hamburg-Eppendorf (UKE) in Germany, the University Health Network in Toronto, Canada, the Ghent University Hospital in Belgium, the Medical University of Warsaw in Poland, and the University of Debrecen in Hungary. The UKE serves as coordinating center and is in charge of the study conduct. Patients will be recruited in routine care but participate from home and data will be collected centrally online by the coordinating center in Germany.

### Participants

To evaluate effectiveness of the intervention, we will include patients with rare autoimmune liver diseases (AIH, PSC, PBC). Besides being diagnosed with one of these conditions, inclusion criteria are a subjective psychosocial support need, an age of at least 18 years, and written informed consent. Exclusion criteria are a life-threatening health status, acute suicidality, ongoing psychotherapy, severe cognitive, auditory or visual impairment, and inability to complete assessments. Peer-counselors are individuals with rare autoimmune liver diseases who feel well adjusted to their disease and strive to help others in the adjustment process, and who provide written informed consent. For the quantitative implementation part, we will include patients who received the intervention, peer-counselors, and healthcare providers involved in intervention delivery. For the qualitative implementation part, we will include four groups of stakeholders: patients/ patient representatives, peer-counselors, healthcare providers, and healthcare leaders such as health insurers, who provide written informed consent. Exclusion criteria are involvement in outcome assessment or data analysis.

#### Sample size

Our primary effectiveness outcome is the group difference in the baseline-adjusted mean score in mental health-related quality of life at post-assessment, assessed with the Short-Form Health Survey (SF-12 [28]). Taking the results of our prior randomized controlled trial (RCT) into account [15], we assume a between groups effect size of *d* = 0.4. Based on two-sided testing with α = 0.05 and 1-β = 0.8, *N* = 100 patients are needed per group (intention-to-treat analysis), yielding in *N* = 200 to be analyzed. Considering our prior RCT [15], we conservatively expect an inclusion rate of 60% and approximately 15% loss to follow-up. Thus, *N* = 240 patients will be included, for which we expect to invite a total of *N* = 400 patients for participation at the five study sites over 15 months.

For the quantitative implementation part of the trial, all patients in the intervention group (*N* = 100), all peer-counselors (~ *N* = 40) and all involved healthcare providers (~ *N* = 25) will be invited to participate in the survey. With an estimated response rate of 80% [15], we expect to include *N* = 132 participants across all sites. At least *N* = 5 participants per stakeholder group and site will be invited to take part in the focus groups, resulting in an estimated sample size of *N* = 100 in total.

### The intervention

The intervention was developed based on pre-assessed psychosocial support needs of patients with rare diseases. A detailed description of the development process has been published [29]. The program is based on structured self-help and peer-counseling. Participants receive a manual, which contains six chapters and which is based on Acceptance and Commitment Therapy (ACT [30]). The first chapter includes general information about rare diseases and a reflection exercise on how the disease affects one’s life. The second chapter focuses on dealing with difficult emotions, the third chapter is about disease acceptance, the forth one about values, the fifth one about setting meaningful goals, and the last chapter contains a review and outlook exercise. Patients complete one chapter per week and, in addition, receive a 30-minute telephone-based peer-counseling session to reflect on the content of the manual. Peer-counselors receive a two-day training, consultation guidelines with additional information and exemplary questions for each chapter as well as supervision by a psychologist or psychiatrist trained in psychotherapy. The regular duration for the program participation is six weeks, but can be expanded to up to ten weeks if any incidents in the patient’s life require a temporary interruption.

### Assessments and outcomes

We will assess quantitative outcomes in both groups at three time points: at baseline (t0), directly after patients in the intervention group completed the program (t1), and at a three-month follow up (t2). Sociodemographic and clinical variables will be assessed at baseline using single items. Qualitative data will be assessed after the follow-up assessment is completed (t3).

#### Effectiveness outcomes

The primary effectiveness outcome is the group difference in mental health-related quality of life at post-assessment, assessed with the Short Form Health Survey (SF-12 [28]). Using 12 items, the SF-12 Health Survey measures psychological and physical aspects of generic, health-related quality of life [31] and is based on the 36-item version SF-36 [32]. Multiple studies demonstrated its sound psychometric properties [33, 34] and it has been used in a variety of chronic conditions, including patients with autoimmune liver diseases [18, 19, 21].

Secondary outcomes are physical health-related quality of life (SF-12), somatic symptom severity (Patient Health Questionnaire-15, PHQ-15 [35, 36]), depression severity (Patient Health Questionnaire-9, PHQ-9 [37, 38]), anxiety severity (Generalized Anxiety Disorder Scale-7, GAD-7 [39, 40]), illness cognitions (disease acceptance, helplessness, perceived benefits; Illness Cognition Questionnaire, ICQ [41]), social support (Social Support Questionnaire, F-SOZU [42]), and self-management abilities (Appraisal of Self-Care Agency Scale Revised, ASAS-R [43]). We will further assess treatment expectations (Treatment Expectation Questionnaire, TEX-Q [44, 45]), psychological burden related to somatic symptoms or associated health concerns (Somatic Symptom Disorder – B Criteria Scale, SSD-12 [46–48]), general self-efficacy (Self-efficacy scale, SWE [49]), and illness perceptions (Brief Illness Perception Questionnaire, B-IPQ [50]).

#### Implementation outcomes

Quantitative implementation outcomes will be assessed at post-assessment (t1). We will ask patients and peer-counselors to evaluate the program and measure acceptability and feasibility on numeric rating scales from 0–10. We will further ask healthcare providers to evaluate the program. Qualitative data will be assessed in focus groups after the follow-up assessment is completed. We will assess perceived implementability of the program into routine care with a standardized semi-structured interview guide. The development of this guide will be supported by the Consolidated Framework for Implementation Research (CFIR) Interview Guide Tool (CFIR Booklet (cfirguide.org)).

### Procedures

Table 1 shows the timeline for patients participating in the study according to the SPIRIT guidelines.

**Table 1:** Timeline according to the SPIRIT guidelines

	**Enrolment**	**Baseline**		**Post- and follow-up**		
**ENROLLMENT**	***-t***_***1***_	**t**_**0**_	**Intervention**	**t**_**1**_	**t**_**2**_	**t**_**3**_
Eligibility screen	X					
Informed consent	X					
Allocation		X				
**INTERVENTIONS**						
Support program			X			
Care as usual	X	X	X	X	X	X
**ASSESSMENTS**						
Sociodemographic variables		X				
Clinical variables		X				
Treatment expectations		X				
**Effectiveness outcomes**						
Mental health-related quality of life		X		X	X	
Physical health-related quality of life		X		X	X	
Somatic symptom severity		X		X	X	
Depression severity		X		X	X	
Anxiety severity		X		X	X	
Self-management abilities		X		X	X	
Illness cognitions		X		X	X	
Social support		X		X	X	
General self-efficacy		X		X	X	
Psychological burden related to somatic symptoms		X		X	X	
Illness perceptions		X		X	X	
**Quantitative implementation outcomes**						
Acceptability, program evaluation					X	
**Qualitative implementation outcomes**						
Program evaluation						X

#### Effectiveness focus

Each partner will recruit eligible patients on a national level by informing them about the study in their routine care. In case this does not suffice to reach the targeted sample size, each partner receives support by a collaborating national patient advocacy organization (PAO), who will recruit nationwide via their homepage, patient journal, newsletter etc. Inclusion and exclusion criteria will be assessed in a structured interview by trained research personnel. Eligible participants will sign an informed consent form. Participants will then be randomly assigned to either the intervention group or a control group. The intervention group will complete the six-week program from home in addition to CAU, whereas the control group will receive CAU alone. After the last data assessment, patients in the control group can participate in the program outside of the trial.

#### Implementation focus

For patients in the intervention group, the quantitative program evaluation will be part of the post-assessment. In addition, stakeholders being involved in delivery of the intervention at each site or in long-term implementation will be invited to take part in the mixed-methods study. They will sign an informed consent form and complete a quantitative cross-sectional survey after the last patient completed the intervention. Qualitative focus groups will be conducted after the follow-up assessment. We pursue a purposeful sampling approach [39] by forming homogenous subgroups with patients/ patient representatives, peer-counselors, healthcare providers, and health insurers in order to describe the particular opinions of these groups in depth.

#### Peer-counselors

Each partner will recruit approximately seven to ten peer-counselors, i.e. individuals with rare liver diseases (PSC, PBC, AIH), who feel well adjusted to their disease and strive to help others in the adjustment process. Their eligibility will be assessed in structured interviews by social science experts (e.g. psychologists) in each team. Peer-counselors then are invited to a two-day training, which will be held in groups of four to five participants at the national study site. After the training, they receive consultation guidelines and supervision. Over the course of the trial, peer-counselors will complete several counseling cycles. Outcomes will also be assessed in peer-counselors before, once during and after their counseling work and they will be invited to participate in the mixed-method process evaluation.

#### Methods against bias

A fixed randomization schedule (allocation ratio 1:1) will be conducted electronically to avoid any preferential patient allocation to the two conditions. Randomization will be conducted by a researcher who is not involved in data assessment and intervention delivery. Neither the patients nor the researchers have any influence on the randomization procedure. *Electronic data collection.* Data will be directly entered into electronic databases by the patients. Thus, error-prone transfer from paper to the electronic databases will be avoided. *Standardization of intervention.* Peer-counselors will be trained and supervised and the intervention is described in peer-counseling guidelines. After each session, peer-counselors will complete a short protocol to assess adherence to the program content. *Blinding.* a) Raters: Outcome assessments will be either self-reported or performed by trained raters who are fully blinded regarding group allocation and not involved in intervention delivery. Thus, Q.RARE.LI is fully observer-blinded. b) Peer-counselors and patients: As in most psychotherapeutic intervention studies, full patient and therapist blinding is not feasible as their active involvement in the intervention is necessary. However, peer-counselors will not be informed about the patient’s group allocation. *Minimization of patient drop-out.* At follow-up, patients will be contacted by telephone according to a schedule of repeated contact attempts. *Standardized report of trial and results.* The trial and the results will be reported according to the CONSORT 2010 recommendations (www.consort-statement.org/).

All data on human subjects will be recorded, handled, and stored by the rules of the General Data Protection Regulation (GDPR). A Data Safety and Monitoring Board (DSMB), independent of the project team and consisting of internationally recognized experts in rare diseases and psychosocial treatments, will oversee the conduct of this study. Before the trial starts, we will prepare a data management plan and all partners will sign a data protection agreement in which they commit to comply to the GDPR.

#### Data collection

The data collection will be organized and supervised by the coordinating center. All quantitative data will be collected electronically into a common database, located at the REDCap system of the University Medical Centre Hamburg-Eppendorf (REDCap, www.project-redcap.org). This will minimize data exchange between countries. Qualitative data will be generated on a national level and securely stored at each site. The focus group discussions will be audio-recorded and transcribed verbatim. The audio records will be deleted after the transcripts have been generated.

### Data handling

Individual participant data will be collected and processed in a de-identified form by a unique study identification number (ID). All data directly identifying persons will be replaced by the study ID. No data allowing participant identification will be handed to third parties. Data collected locally from the individual partners (original consent forms, audio files, and interview transcripts) will be securely archived at the specific sites for maximum 10 years after publication of the last scientific report, following the national data management strategy. If locally collected data is exchanged between partners, then only in fully anonymized form. For locally collected data, compliance with the GDPR will be determined in the data protection agreement. In accordance with the data minimization principle, we will only collect data that is relevant to the purposes of the project.

### Data analysis

We will calculate descriptive measures of all variables and examine group differences at baseline. Our primary hypothesis within the RCT will be tested with a hierarchical linear model including time (two-staged) and group as factors, time as repeated effect, the time x group interaction, the baseline outcome score as a covariate, and a random intercept. We will use the restricted maximum likelihood method to produce estimates. To assess group differences for each time point, we will determine estimated marginal mean values (EMMeans). Effect sizes will be calculated by dividing the adjusted group mean difference by the observed standard deviation of the total sample at baseline. Model assumptions will be checked by plotting residuals against estimated values and residual distribution against normal distribution. We will perform all tests two-sided and consider *p* < 0.05 as statistically significant. Data will be imputed if more than 5% are missing. In accordance with White et al. [51], the number of imputations will be chosen depending on the proportion of missing data.

Quantitative data will be analyzed descriptively (mean, standard deviation, percentages, figures). Qualitative data will be analyzed with qualitative content analysis according to Mayring [52]. We will deductively derive implementation barriers and facilitators based on the CFIR (www.cfirguide.org), i.e. considering the outer and inner setting, intervention characteristics, individuals involved, and the process. Based on these contextual conditions, we will derive implementation strategies with the CFIR.

#### Data sharing

In accordance with the ethics committee approval and the 2015 German Research Foundation (DFG) guidelines for the handling of research data, the quantitative individual patient data will be made publicly available in a de-identified form. The full data package (i.e. analyzable data set, protocol, statistical analysis plan, statistical programming code) will be made freely available through a clinical data repository (e.g. Dryad Digital Repository) and saved for at least 10 years. Data sharing will follow the FAIR Data Principles (Findable, Accessible, Interoperable, and Reusable) and international naming conventions (e.g. Systematized Nomenclature of Medicine) to maximize transparency and scientific reproducibility.

### Ethical aspects

All research activities in Q.RARE.LI will respect fundamental ethics principles, including those reflected in the Charter of Fundamental Rights of the European Union and the WMA Declaration of Helsinki, and comply with international, EU, and national laws. Each partner will obtain an ethics approval by an independent local ethics committee (see Table**2** in the declarations section for an overview). The coordinator will ensure compliance with ethical principles and is advised by the data protection officer of the UKE.

**Table 2:** Overview of ethics approvals

Partner	Name of ethics committee	ID	Status
Germany, coordinating center	Independent Ethics Committee of the Hamburg Medical Chamber, Weidestr. 122 b, 22,083 Hamburg, Germany Phone: + 49 40 202,299–240, E-mail: ethik@aekhh.de	2021–100,757-BO-ff	Approved January 31^st^, 2022
Canada	University Health Network Research Ethics Board, 700 University Ave, 4th Floor, Toronto, Ontario, M5G 1Z5 Phone: (416) 581–7849	22–5056	Approved February 14^th^, 2023
Belgium	Committee on Medical Ethics, UZ Gent, Corneel Heymanslaan 10, 9000 Ghent, Belgium Phone: + 32 9 3,322,111, E-mail: ethisch.comite@uzgent.be	BC-10401	Approved February 22^nd^, 2023
Poland	The Local Ethics Committee of Medical University of Warsaw, ul. Pawińskiego 3C, 02–106 Warszawa, Poland, Phone: + 48 22 57 20 303, E-mail: komisja.bioetyczna@wum.edu.pl	KB/26/ 2022	Approved February 21^st^, 2022
Hungary	Medical Research Council, Scientific and Research Ethics Committee (ETT TUKEB), 25 Alkotmány u., Budapest, H-1054, Hungary, Phone: (+ 36 1) 795 1192, E-mail: attilane.gombos@bm.gov.hu	40,513–5/2021/EÜIG,	Approved August 16^th^, 2021

#### Informed consent

Before inclusion, eligible participants are informed about the study procedure and data protection verbally and in written form by the local research team. They are also informed that consent to participate is voluntary and can be withdrawn at any time without giving reasons, and without any disadvantages. Written information will be provided in participants’ local language and checked by a patient representative regarding comprehensibility beforehand. Written informed consent forms will be signed by all participants and sent to the local research team. Participants will receive a financial compensation for their participation in the study.

#### Risks and benefits

In general, psychosocial support interventions bear no major risk for severe adverse events. In our efficacy trial [15], no major adverse events occurred and no individual stated that the intervention harmed them. Rather, participating in the intervention was beneficial for patients. Being involved as a peer-counselor can potentially be beneficial, too [53]. However, adverse events unrelated to the intervention may occur. If suicidal ideation is detected (e.g. during selection interviews or in peer-counseling sessions), we will apply a proven algorithm (e.g. contact the physician or consider psychiatric treatment) that is already available from other studies of the project team. Before the trial, the staff (including peer-counselors) will be carefully advised to follow these guidelines and provided with an emergency number. In cases of acute suicidality, the patient will be transferred to receive psychiatric treatment and excluded from the study. Any questions regarding patient exclusions, serious adverse events, and potential study termination will be reported to and reviewed by the DSMB. The DSMB will annually monitor the study and evaluate the collected study data with regard to participant safety, study conduct, compliance with the study protocol and progress. Where appropriate, recommendations will be made to continue, modify, or terminate the study or to unmask participants in case of adverse events. For the individual patient, the trial procedure will stop if any adverse events or withdrawal of informed consent occur. The whole trial will be discontinued if the consortium or the DSMB detect significant associations between study participation and adverse events. 

### Dissemination

The results will be published and disseminated nationally and internationally to the scientific community as well as to the patient community and public. The main findings will be submitted for publication in a high-impact peer-reviewed journal with open-access mechanism within 12 months of study completion. Authorship eligibility will be decided based on the International Committee of Medical Journal Editors (ICMJE). We will also regularly communicate the results in lay language via press releases, social media, our own homepages and forums which are popular amongst patients. The close collaboration with PAOs in each participating country ensures adequate communication to the patient community via newsletters and yearly events, which we plan to attend in order to present the results.

### Patient involvement

The intervention has been developed based on pre-assessed needs of patients with different rare diseases [29] and was supported by the German patient organization ACHSE e.V. Within Q.RARE.LI, patients are actively involved as stakeholders when evaluating the implementability of the intervention into routine care. Moreover, the engagement of peer-counselors meets the need of a greater patient empowerment and has been formulated by the Word Health Organization as a strategy to improve care [54].

## Discussion

Q.RARE.LI aims to pave the way for widely available psychosocial support for the rare disease community. We expect to demonstrate the effectiveness of the peer-delivered psychosocial support intervention in routine care of patients with rare autoimmune liver diseases in five different national health systems. With respect to implementation outcomes, we expect high acceptability and perceived feasibility of the intervention from the perspective of patients, peer-counselors, and healthcare providers. In addition, we expect to gain a deep understanding of the implementation context in five different countries and be able to derive barriers and facilitators for implementation based on the CFIR framework. This will lead to a profound understanding of the intervention characteristics, the outer setting, the inner setting, the individuals involved as well as the process from different perspectives (including patients and healthcare providers). Identifying these contextual determinants will help us to derive appropriate implementation strategies and develop a concrete implementation plan in each country. If our hypotheses are confirmed, we will be one step closer to making targeted psychosocial support available to a large group of patients with rare diseases. Independent of the effectiveness results, the results on implementation will lead to a better understanding of the implementation conditions in five different healthcare settings and facilitate future implementation of any psychosocial support program.

With Q.RARE.LI, we pursue a transdiagnostic approach in psychosocial care. Considering the high clinical heterogeneity between different rare diseases, even among conditions of the same group such as autoimmune liver diseases, this may seem counterintuitive. However, the transdiagnostic approach offers a major advantage in psychosocial care of this patient population. At a structural level, cross-disease services are what makes comprehensive psychosocial care possible in the first place as offering disease-specific services for the multitude of different rare diseases is considered impossible [13]. At a content level, this approach is adequate as well. Despite being affected by heterogeneous clinical symptoms, patients with different rare diseases experience a variety of similar problems, including psychological burden, constraints in everyday and social life, and problems related to the healthcare system [3]. Addressing these in transdiagnostic psychological interventions can help to reach many individuals within this hard-to-reach patient population for psychosocial support.

## Data Availability

After publication of the results, de-identified individual patient data will be made publicly available through a clinical data repository. Contact person: Dr. Natalie Uhlenbusch, n.uhlenbusch@uke.de.
